# Case of Puerperal Convulsions Treated by Tracheotomy

**Published:** 1854-05

**Authors:** Marshall Hall


### BUFFALO MEDICAL JOURNAL

AND

### MONTHLY REVIEW.

**VOL. 9.** MAY, 1854. **NO. 12.**

### ORIGINAL COMMUNICATIONS.

ART. I.—*Case of Puerperal Convulsions treated by Tracheotomy.* Communicated by Dr. MARSHALL HALL.

To the Editor of the Buffalo Medical Journal.

SIR:—I think the following case will not be without interest for your readers. It is to be regretted that the orifice made into the trachea was not of more ample dimensions. To prove availing it should be sufficient to remove *all* laryngeal dyspnœa during the paroxysms of convulsions, absolutely. It should also have been performed earlier, perhaps instead of the second blood-letting.

I am, Sir,

Your obed't servant,

MARSHALL HALL.

BUFFALO, April 3, 1854.

Mrs. G——, a delicate woman, aged 29, was roused from her sleep at 3, A. M., March 7, by the escape of the liquor amnii, which was the first notice she received of the commencement of labor, having arrived at the full period of a first pregnancy; all seemed to progress favorably until about half-past nine; vomiting, yawning, and headache being then present. During this period her countenance was unusually pale, but these symptoms did not create alarm in the mind of the gentleman in attendance, until 9.30, when a most severe and sudden fit of convulsions attacked her, which lasted about an hour, in which *the face was very livid, and suffused, and the veins of the head and neck much distended, the carotids beating most violently.* The general convulsions and contraction of the countenance, were of the most fearful description. On my arrival a vein in each arm was opened, and finding the fits returning with great violence at short intervals, I resolved to empty the uterus as speedily as possible, and I practiced craniotomy and removed the fœtus. The placenta was expelled, by uterine action, in about five minutes; there was no hæmorrhage; and the convulsions subsided. Consciousness was restored, and some gruel taken. She remained in this state for about an hour, when the convulsions set in again most violently. The jugular vein was opened, as well as a vein in the arm; the hair cut off, ice applied to the head, hot applications to the feet, cataplasms, &c., &c., &c. Terebinthinate enemata were administered; still convulsions returned, and I could but reflect upon the parallelism (to my mind) between this case and the epileptic cases in which Dr. Marshall Hall has recommended the adoption of tracheotomy; and as asphyxia was threatened *every moment*, and the constriction of the neck, and distended veins, &c., made each attack so perilous, I resolved to operate, though in the middle of the country, and without the usual appliances for so delicate an operation, which, also, was rendered more difficult by an enlarged thyroid gland. I, however, converted the silver tube of my caustic case (the end being removed) into a tracheal pipe secured by a tape, and with the aid of the few things my pocket case contained in addition to my penknife, I accomplished my purpose. Not a dessert-spoonful of fluid was lost, and *the moment the trachea was opened the relief was apparent in the countenance, which never after became in the least livid, neither did the veins ever become distended.* The fits did not, however, cease, but they became *less frequent*, and *less violent*, and it was evident that there was a marked diminution of suffering to the patient, and time given for the administration of remedies, which, however, ultimately proved of no avail, for the patient sank about noon the next day, having survived delivery about twenty-four hours.

I should mention that her case was complicated with bronchitis, having, a fortnight before, taken cold and been coughing a good deal, and the quantity of muco-purulent secretion discharged from the operation was very great. There is also another circumstance with which I was struck: the absence of convulsions during the time the cataplasms were smarting (while consciousness remained) as well as during the pain of the operation and the passage of the child and after-birth into the world. The first convulsion immediately followed a digital examination. Her nervous system had received a great shock about twelve days before which had affected her very much. The convulsions ceased about 1 o'clock in the morning, and a state of coma continued to the last.

